# General Growth of Carbon Nanotubes for Cerium Redox Reactions in High-Efficiency Redox Flow Batteries

**DOI:** 10.34133/2019/3616178

**Published:** 2019-11-11

**Authors:** Zhaolin Na, Ruifang Yao, Qing Yan, Xudong Sun, Gang Huang

**Affiliations:** ^1^Liaoning Engineering Laboratory of Special Optical Functional Crystals, College of Environmental and Chemical Engineering, Dalian University, Dalian 116622, China; ^2^Institute of Ceramics and Powder Metallurgy, School of Materials Science and Engineering, Northeastern University, Shenyang, Liaoning 110819, China; ^3^WPI Advanced Institute for Materials Research, Tohoku University, Sendai 980-8577, Japan

## Abstract

Carbon nanotubes (CNTs) possess remarkable mechanical, electrical, thermal, and optical properties that predestine them for numerous potential applications. The conventional chemical vapor deposition (CVD) route for the production of CNTs, however, suffers from costly and complex issues. Herein, we demonstrate a general and high-yield strategy to grow nitrogen-doped CNTs (NCNTs) on three-dimensional (3D) graphite felt (GF) substrates, through a direct thermal pyrolysis process simply using a common tube furnace, instead of the costly and complex CVD method. Specifically, the NCNTs-decorated GF (NCNT-GF) electrode possesses enhanced electrocatalytic performance towards cerium redox reactions, mainly due to the catalytic effect of N atoms doped into NCNTs, and ingenious and hierarchical 3D architecture of the NCNT-GF. As a result, the cell with the NCNT-GF serving as a positive electrode shows the improved energy efficiency with increases of about 53.4% and 43.8% over the pristine GF and the acidly treated GF at a high charge/discharge rate of 30 mA cm^−2^, respectively. Moreover, the as-prepared NCNT catalyst-enhanced electrode is found to be highly robust and should enable a long-term cycle without detectable efficiency loss after 500 cycles. The viable synthetic strategy reported in this study will contribute to the further development of more active heteroatom-doped CNTs for redox flow batteries.

## 1. Introduction

Large-scale energy storage is envisioned as a key technology for improving sustainability in the electricity generation sector, by circumventing the intermittency and uncertainty of renewable energy, offering regulatory services, and increasing the efficiency of the existing fossil fuel infrastructure [1, 2]. Particularly, the redox flow battery (RFB) has emerged as an attractive device for grid storage due to its desirable advantages that include a long service life, capability of a deep discharge, and rapid response to load change. As compared with other conventional secondary batteries, the most attractive attribute of a RFB is that energy capacity and power output are decoupled from each other, with the energy capacity depending on the electrolyte concentration and volume while the power output is determined by the electrode size [3–6]. A variety of RFB chemistries have been researched in recent years such as Br-S [7], Fe-Gr [8], and Zn-Br [9]. In the myriad chemistries employed in RFB systems, the all-vanadium RFBs (VRBs) have gained much attention attributed to their high electrochemical reversibility, long life, and lower risk of cross-contamination [10–13]. Nevertheless, the drawbacks of VFBs including the high initial installation cost, relatively high toxicity, low standard cell voltage (1.26 V), and the resulting energy density (c.a. 20-30 W h L^−1^) are still main hindrances to the broad market penetration of this technology [14]. Alternative redox species/couples could alleviate some of these issues. Increasing the operating voltage window can be achieved using a redox reaction that has a more positive redox potential in a positive electrolyte [15]. Specifically, the Ce(IV)/Ce(III) couple with a potential up to 1.72 V *vs.* SHE [16] was used as a positive active material in RFB designs (e.g., Ce-Zn [17–23], Ce-V [14], and Ce-H [24, 25] batteries) to achieve a higher cell voltage. Also, our groups presented the properties of novel cerium-lead systems [26–28] which employ the Ce(IV)/Ce(III) and Pb(II)/Pb redox reactions:

(i) Positive electrode
  (1) $$\begin{array}{c} 2\text{Ce}\left(\text{III}\right)\overset{\text{charge}}{\underset{\text{discharge}}{⇄}}2\text{Ce}\left(\text{IV}\right)+2{\text{e}}^{-}\left(E^{0}=+1.72\text{V }vs.\text{SHE}\right) \end{array}$$
(ii) Negative electrode
  (2) $$\begin{array}{c} \text{Pb}\left(\text{II}\right)+2{\text{e}}^{-}\overset{\text{charge}}{\underset{\text{discharge}}{⇆}}\text{Pb }\left(E^{0}=-0.13\text{V }vs.\text{SHE}\right) \end{array}$$
(iii) Overall cell reaction
  (3) $$\begin{array}{c} 2\text{Ce}\left(\text{III}\right)+\text{Pb}\left(\text{II}\right)\overset{\text{charge}}{\underset{\text{discharge}}{⇄}}\text{Pb}+2\text{Ce}\left(\text{IV}\right)\left({E^{0}}_{\text{cell}}=1.85\text{V }vs.\text{SHE}\right) \end{array}$$

The cerium-lead RFB has several desirable attributes for large-scale energy storage as follows. The great advantage of this RFB is its high thermodynamic cell potential (1.85 V) which is among the highest of aqueous RFBs [21]. Moreover, the costs of required reagents are moderate and their chemistry has significantly less toxicity. In addition, superior electrochemical performance can be successfully acquired for these batteries in extended charge/discharge operations under a broad temperature range from -20°C to 40°C [26].

Notwithstanding, the increased overall cell polarization and resulting declined cell performance, especially at a higher charge/discharge rate, inevitably impedes the achievement of better rate capacity and efficiency. The electrode is a crucial component of the RFB as it provides electroactive surfaces and conducts electrons for the respective electrochemical reactions to take place. Hence, catalytic activity, wettability, and mass transport properties of the electrode materials directly determine RFB performance [29]. And as for the RFB, the ideal electrodes should offer both long-term durability and stable catalytic activity. Currently, the most commonly used material for RFB electrodes is the commercially available graphite felt (GF) because of its advantages including wide operating electrode potential range, porous structure beneficial for electrolyte flow, and reasonable price [30, 31]. However, the low electrochemical activity and poor reversibility of the pristine GF restrict its extensive application in RFBs [6]. Therefore, various approaches have been introduced to enhance electrocatalytic properties of GFs, through the deposition or incorporation of nanostructured electrocatalysts onto the GF surface. Generally, there are two categories of electrocatalysts including metal-based (noble metal [32–34] and metal oxide [27, 35–40]) and carbon-based nanomaterials [41–43] for enhancing the electrochemical activity of GFs. Precious metal-based catalysts have shown satisfactory catalytic performance towards vanadium redox reactions, whereas the high cost and the gas coevolution issue related to them represent a limit for a practical application [33]. An alternative approach to develop the high-performance electrodes with reasonable cost and high stability in acid conditions is to introduce carbon-based nanomaterials.

Among carbon materials, one-dimensional (1D) carbon nanotubes (CNTs) have been widely used in electrochemical applications such as hydrogen storage, fuel cells, supercapacitors, and lithium-ion batteries owing to their unique electrical and structural properties [44–46]. CNTs have also served as good electrode materials for vanadium redox couples in VFB systems. The case of growing CNTs on the fiber surface of GFs does enhance the performance of VFBs [42, 47, 48]. The most common approach to the synthesis of CNTs is chemical vapor deposition (CVD) [49]. Through using different variations of the CVD method, GF electrodes can be successfully modified with CNTs [50], CNTs/CNFs (carbon nanofibers) [47], and graphene nanowalls [51] and the resulting increase in the specific active surface area is observed to enhance the electrochemical performance of all-vanadium RFBs in all cases. Unfortunately, the CVD synthesis process is costly and complex, and there is a crying need to develop a simple approach to prepare such CNT catalysts with high activity and stability [52]. What is more, the utilization of CNTs as electrocatalysts for cerium-based RFBs has not been investigated yet.

Herein, we design a generalizable and high-yield strategy for the successful growth of N-doped CNTs (NCNTs) on GFs through a direct thermal pyrolysis process using a common tube furnace (see [Supplementary-material supplementary-material-1] in Supplementary Materials for detail regarding the device fabrication), instead of the costly and complex CVD method. Porous GFs can act as ideal substrates that provide a brilliant porous framework for NCNT growth. The as-prepared architecture possesses a large active surface area, stable frameworks, controlled dopants, and enriched pores, thus resulting in high electrocatalytic kinetics and reversibility towards Ce(VI)/Ce(III) redox couples. Consequently, when applied in cerium-based RFBs, the proposed NCNT-decorated GF (NCNT-GF) exhibits excellent performance as RFB electrode materials. The results from systemic structural analysis and electrochemical measurements demonstrate that the synergistic effect between the hierarchical 3D architecture of the NCNT-GF and appropriate N doping endows the NCNT-GF with outstanding electrochemical property. This finding gives further insights into the structure-property correlation of heteroatom doping in NCNTs for cerium-based RFB applications.

## 2. Results and Discussion

**Scheme 1:**
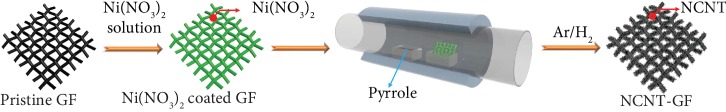
Illustration of the fabrication process of NCNT-GF.

The overall synthetic procedure for NCNT-GF, simply using a common tube furnace instead of the costly and complex CVD method, is illustrated in Scheme 1 (see Materials and Methods and [Supplementary-material supplementary-material-1] in Supplementary Materials for detail regarding the device fabrication and material preparation). Firstly, the GF was immersed into the as-prepared nickel nitrate solution and subsequently dried at 80°C for 5 h. To grow the NCNT catalyst on GF, the dried GF and pyrrole were put into two separated ceramic boats in a quartz tube with pyrrole at the upstream side of the tubular furnace ([Supplementary-material supplementary-material-1]). Then, the sample was calcined at 800°C under a static Ar/H_2_ (5% H_2_) atmosphere and subsequently naturally cooled to ambient temperature. After the sequential removal of the metal impurities with concentrated sulfuric acid, the NCNT-decorated GF (NCNT-GF) electrode is obtained. This method is very simple in that it does not require any costly and complicated equipment and steps, where a common tube furnace serves as the device for the material preparation. During the pyrolysis process, the pyrrole acts as a single precursor for the preparation of NCNTs, while the Ni species serve as a growth catalyst by reducing the energy barrier to form NCNTs and promoting their growth.

**Figure 1:**
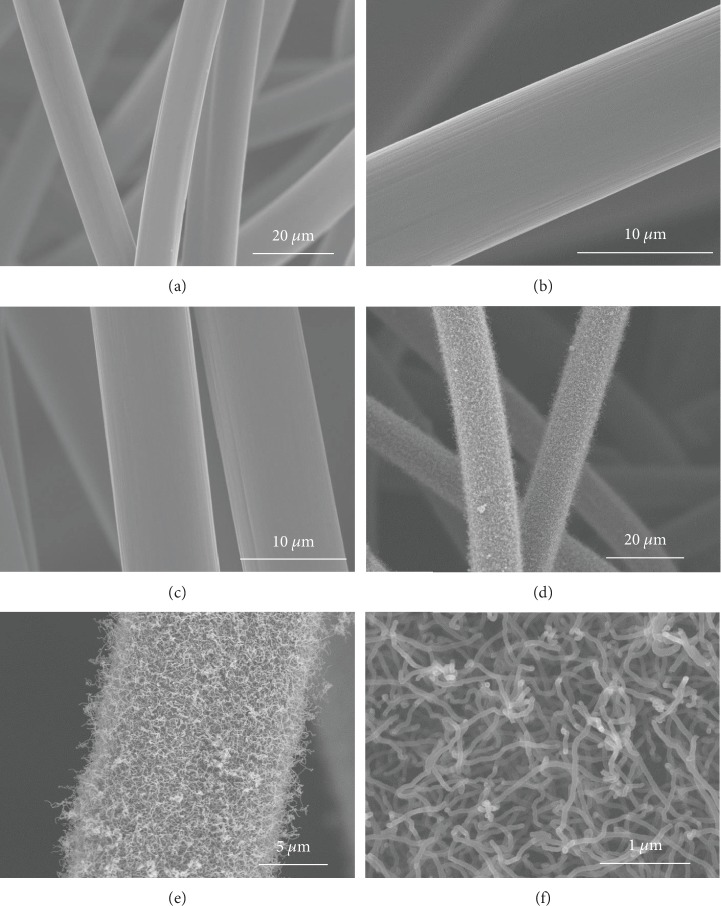
SEM images of (a) GF, (b, c) A-GF, and (d–f) NCNT-GF electrodes.

The scanning electron microscopy (SEM) images of the as-prepared samples are shown in Figure 1. It can be observed in Figure 1(a) that the pristine GF consists of numerous carbon fibers with 3D porous structure and then displays a smooth surface, which would favor the mass transport of electrolytes. As we all know, the wettability of the electrode material would show great effect on the RFB performance [27]; therefore, the pristine GF is subjected to concentrated sulfuric acid oxidation to enhance the wettability and so enlarge the active specific surface area. After acid activation, as presented in Figures 1(b) and 1(c), the A-GF prepared through acid activation exhibits a clean and smooth surface similar to the pristine GF suggesting a minor structural degradation by oxidation treatment. The rough surface of GF after immersing into nickel nitrate solution in [Supplementary-material supplementary-material-1] shows that nickel nitrate can be successfully impregnated onto the GF surface through the immersion-dry method. Energy dispersive X-ray spectroscopy (EDS) elemental mapping images further demonstrate that nickel nitrate is uniformly distributed on the GF surface ([Supplementary-material supplementary-material-1]). As clearly displayed in Figures 1(d) and 1(e), after thermal pyrolysis treatment, the carbon fiber surface is uniformly covered by a randomly oriented, entangled NCNT forest due to the well-dispersed Ni-based catalyst, and the NCNT-GF hybrid shows a well-interconnected network structure. The diameter of the NCNTs is around 47 nm with an approximate length of around several to a few tens of micrometers, as displayed in Figure 1(f). The diameter of the NCNTs is considerably smaller relative to GF fibers (about 10 *μ*m), leading to an improved specific surface area for the electrochemical reactions.

**Figure 2:**
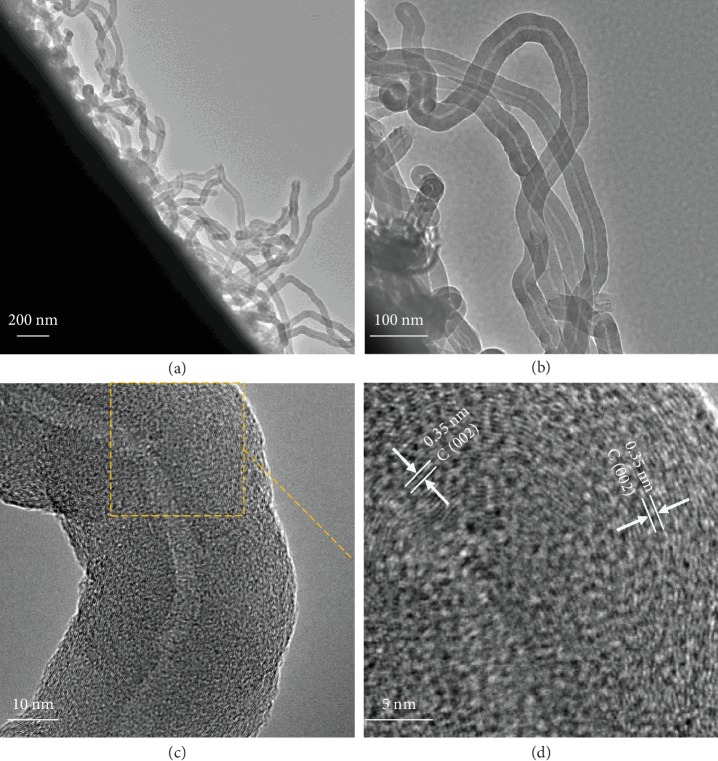
(a, b) TEM and (c) HRTEM images of the synthesized NCNT-GF. (d) HRTEM lattice images taken from the framed region in part (c).

The low-magnification TEM image of NCNT-GF further confirms the successful introduction of NCNTs onto the GF surface and the formation of the tubular structure, as shown in Figure 2(a). [Supplementary-material supplementary-material-1] shows a representative TEM image of NCNT without any posttreatment after the growth process. It can be clearly observed that the catalyst nanoparticles (Ni, decomposed from nickel nitrate) for NCNT growth still exist in the as-grown NCNT. Simple immersion of the as-prepared sample into concentrated sulfuric acid can effectively remove the Ni nanoparticles, as evidenced in Figures 2(a) and 2(b). More detailed observation by high resolution transmission electron microscopy (HRTEM) (Figure 2(c)) indicates that these thin NCNTs directly grown on GF have outer diameters ranging from 40 to 50 nm and inner diameters of ∼8 nm, showing a multiwalled feature. Small graphitic layers with d-spacing of 0.35 nm (corresponding to (002) crystalline planes of graphite [52]) are randomly stacked in the walls and not parallel to the axis direction, exhibiting more defects and edges in NCNTs that can serve as active sites for cerium redox reactions (Figure 2(d)). It is evident from the SEM and TEM micrographs that the CNTs grown by this strategy are more dense and uniform, and the size distributions are relatively more homogenous, compared to the recently reported CNTs grown by CVD [53–55].

**Figure 3:**
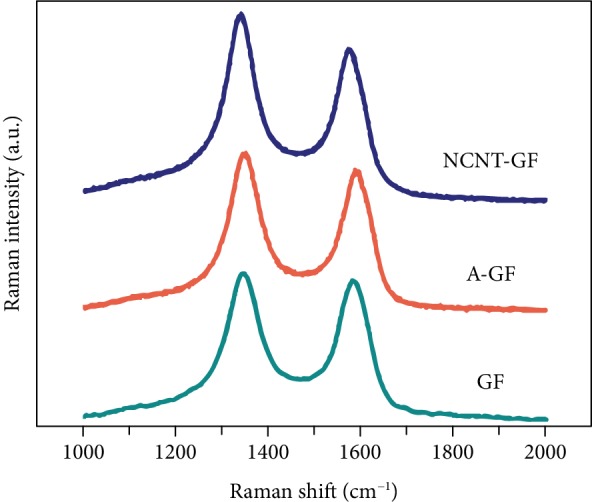
Raman spectra of GF, A-GF, and NCNT-GF electrodes.

Raman spectroscopy is used to further characterize the microstructures of the electrode samples. As indicated in Figure 3, two well-defined peaks can be clearly appreciated at around 1341 and 1583 cm^−1^ which correspond to D and G bands, respectively, for all samples. It is well-known that the D band represents structural disorder, which is characterized by bond angle distribution and linked with sp^3^ carbons, and that the G band corresponds to the *E*_2g_ symmetric vibrational mode of graphite type sp^2^ carbons [56]. Commonly, the intensity ratio between the D and G bands (*I*_D_/*I*_G_) is used as a qualitative indicator of the defect density in carbon samples. The *I*_D_/*I*_G_ ratio for the NCNT-GF sample (1.312) is much higher than those for GF (1.099) and A-GF (1.167). The increased *I*_D_/*I*_G_ band intensity ratio in the NCNT-GF reveals large amounts of highly electrochemically active defects and edge plane exposure, consistent with the TEM observations. This can be ascribed to the heterogeneous nitrogen atom doping onto the graphite layers of NCNTs.

**Figure 4:**
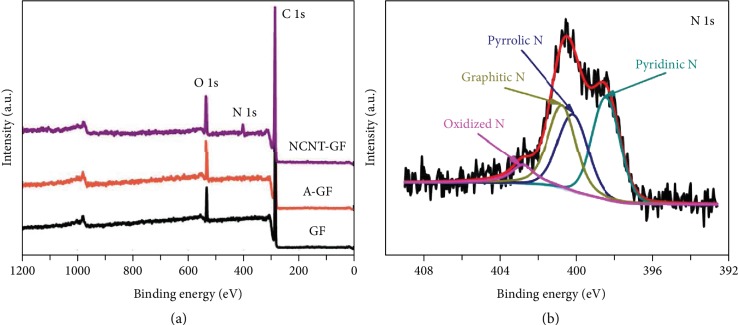
(a) Survey XPS spectra of GF, A-GF, and NCNT-GF. (b) XPS N 1s spectrum of NCNT-GF.

X-ray photoelectron spectroscopy (XPS) is conducted for elucidating the chemical composition and nitrogen-bonding configuration in the NCNT-GF. As shown in Figure 4(a), no significant signals can be found in the N 1s region in the survey spectra of the GF and A-GF samples, while the N 1s peak is very significant for NCNT-GF. This confirms the successful incorporation of N atoms into the carbon lattice of CNTs. The XPS spectra also confirm that all samples are free from metal impurities of Ni (<0.1 at.% detection limit), as further supported by TEM investigation. The atomic contents of C, N, and O in different samples according to XPS analysis are specified in [Supplementary-material supplementary-material-1]. The N content in the NCNT-GF is measured to be around 4.90 at.%, while the O content of NCNT-GF (about 8.25 at.%) is lower than that of A-GF (about 11.48 at.%). The N 1s spectrum of NCNT-GF can be deconvoluted into four peaks (Figure 4(b)) at 398.4, 400.0, 400.8, and 402.8 eV, corresponding to the pyridinic nitrogen, pyrrolic nitrogen, graphitic nitrogen, and oxidized species of nitrogen, respectively [57]. The molecular bonding structures of these nitrogen functional groups can be described as follows. Pyridinic N refers to the nitrogen atom that bonds to two carbon atoms and donates one p-electron to the aromatic *π* system. Graphitic N is a type of nitrogen that is incorporated into the plane of the graphene matrix and bonds to three carbon atoms. Pyrrolic N is the nitrogen atom that is incorporated into five-membered heterocyclic rings and contributes to the system with two p-electrons. Oxidic N is a nitrogen atom bonded with two carbon atoms and one oxygen atom. Such nitrogen species can deform the structure of NCNTs and introduce lots of defect sites in CNT lattices [52], as suggested by the Raman data (Figure 3). These defects can work as the active sites for the oxidation and reduction of cerium species (electrons are transferred between reactants and electrodes), leading to improved electrochemical activity, thus boosting the electrocatalytic activity of N-doped carbon samples. Besides, according to density functional theory (DFT) calculations, carbon atoms adjacent to nitrogen dopants possess a substantially high positive charge density to counterbalance the strong electronic affinity of the nitrogen atom [58–61]. Hence, the nitrogen-induced charge delocalization would facilitate the adsorption process of positively charged reactant ions onto the electrodes and contribute to the ion exchange during the redox reaction [62].

**Figure 5:**
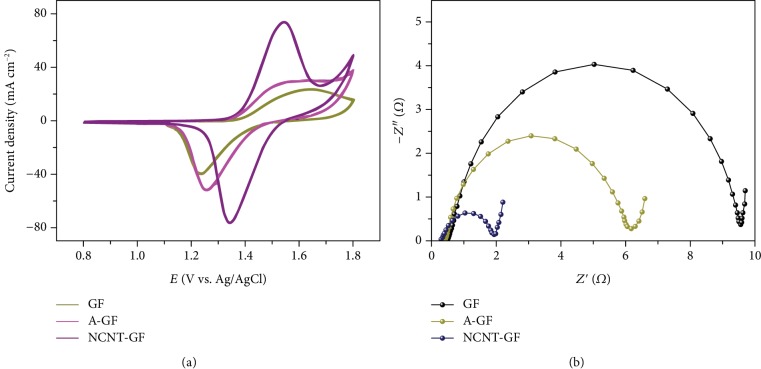
(a) CV curves on different electrodes at 3 mV s^−1^. (b) Nyquist plots of various electrodes.

Cyclic voltammetry (CV) is performed on pristine graphite felt and functionalized graphite felt electrodes to estimate their electrochemical activity towards the Ce(IV)/Ce(III) redox reaction. As displayed in Figure 5(a), two peaks are exhibited in each curve, corresponding to the redox reaction of Ce(IV)/Ce(III) couples. The result suggests that the Ce(IV)/Ce(III) couple reaction exhibits a characteristic of quasireversion on all samples. The GF electrode shows an anodic (*I*_pa_) peak current density of 24.0 mA cm^−2^ and a cathodic peak current density (*I*_pc_) of 39.3 mA cm^−2^. A-GF shows a rise of *I*_pa_ up to 30.4 mA cm^−2^ and *I*_pc_ up to 51.4 mA cm^−2^, compared to GF. And as for NCNT-GF, the values of *I*_pa_ and *I*_pc_ further rise up to 74.2 and 75.9 mA cm^−2^, respectively. The remarkably higher peak current densities on NCNT-GF imply more favorable electron transfer kinetics in cerium redox reactions, compared with those on GF and A-GF. The peak potential difference (DE_p_) and the ratio (*I*_pa_/*I*_pc_) which can be obtained from Figure 5(a) are two key criteria in the reversibility evaluation of the redox reaction. The value of DE_p_ on NCNT-GF is 202 mV, much smaller than the DE_p_ values on the A-GF and GF (381 and 410 mV, respectively) at the same experimental conditions. In addition, the values of *I*_pa_/*I*_pc_ for NCNT-GF, A-GF, and GF are 0.98, 0.59 and 0.61, respectively. Combining the values of DE_p_ and *I*_pa_/*I*_pc_, it could be concluded that the NCNT-GF electrode affords a better reversibility for the redox reaction of Ce(III)/Ce(IV) redox pairs relative to the GF and A-GF.

For further investigation of the electron transfer process, electrical impedance spectroscopy (EIS) is performed, and the corresponding Nyquist plots are shown in Figure 5(b). The arc of the Nyquist plots in the high-frequency range is associated with charge transfer reactions at the electrolyte/electrode interface, while the straight line with a unique slope at the low frequency range reflects the diffusion process. This implies that the Ce(IV)/Ce(III) redox reaction is a mixture of kinetic and diffusion-controlled processes on all samples. The radius of the semicircle reflects the value of the charge transfer resistance at electrolyte/electrode interfaces, and a smaller radius implies faster reaction kinetics [63]. From the results shown in Figure 5(b), it can be observed that the resistance related to the charge transfer reaction decreases in the following order: GF > A − GF > NCNT − GF, which is in agreement with the results of the CV measurements. The enhanced electron transfer rate of NCNT-GF may stem from the N dopant, similar to the O dopant in A-GF [47], accelerating the electron transfer rate for the cerium redox reactions. This result indicates that the NCNT-GF electrode indeed largely boosts the rate of Ce(IV)/Ce(III) redox reaction.

On the basis of CV curves at different scan rates, the mass transfer features for the as-prepared electrode can be assessed by plotting the peak current density versus the square root of scan rate [45]. [Supplementary-material supplementary-material-1] show the CV profiles of the GF, A-GF, and NCNT-GF measured at various sweeping rates (3, 5, 7, and 10 mV s^−1^). The cathodic peak current is proved to be nearly proportional to the square root of the scan rate on all three samples, while it can be clearly observed that the slope of NCNT-GF is much steeper than those of GF and A-GF, indicating a faster mass transfer process on the surface of NCNT-GF. The distinctly faster mass transfer rate on NCNT-GF might stem from the enhanced hydrophilic property and lower surface energy induced by the nitrogen doping. Given that the diffusion coefficient for the active ions in electrolytes depends on electrolyte properties, a higher apparent diffusion rate must be because of a higher effective surface area for NCNT-GF in addition to the 3D porous property of GF, which facilitates the mass transport behavior at electrode/electrolyte interfaces. The electrochemical surface area (ECSA) can determine the area linked to the conductive path available to transfer electrons to/from the surface of the electrode. And the ECSA can mirror the number of the electrocatalytically active sites that is available for electrochemical reactions. The ECSA of the electrodes can be assessed using the Randles-Sevcik equation [64] (see detail from Supplementary Materials [Supplementary-material supplementary-material-1]). On the basis of the slope for each electrode ([Supplementary-material supplementary-material-1]), the obtained ECSA values (cm^2^) increase in the following order of GF (131), A-GF (138), and NCNT-GF (219). This supports the fact that the NCNT-GF can supply more well-developed electrochemically active sites for Ce(IV)/Ce(III) couple reactions relative to the other samples. This is probably due to the fact that a great number of defect sites are generated through N doping in the preparation process, as supported by TEM observations. As a consequence, the improved effective surface area is responsible for the higher mass transfer rate and enhanced kinetics at electrolyte/electrode interfaces.

In addition, the standard rate constant, *k*_0_, could be estimated by the ln (*i*_*p*_)*vs.E*_*p*_‐*E*^0^ plot ([Supplementary-material supplementary-material-1]) based on the following equation [65], which correlates the intrinsic reaction kinetics on the surface of electrode samples, to the peak potential separation and peak current (for more details, see Supplementary Materials [Supplementary-material supplementary-material-1]):

(4) $$\begin{array}{c} i_{p}=0.227{nFAC}_{0}k_{0}\exp\left[\frac{-\alpha nF\left(E_{p}-E^{0}\right)}{RT}\right]. \end{array}$$

A dramatic increase of *k*_0_ for the cerium redox reaction on different samples could be in the order of GF (9.92 × 10^−7^ m s^−1^) < A − GF (6.84 × 10^−5^ m s^−1^) < NCNT − GF (2.09 × 10^−4^ m s^−1^). Notably, the NCNT-GF exhibits a remarkably higher rate constant and thus a significantly enhanced electrocatalytic performance relative to GF and A-GF, matching well with CV and EIS results. The remarkably improved electrocatalytic performance of NCNT-GF may stem from the N dopant, which can catalyze the cerium ion redox reactions through generating active sites for these reactions. Compared to the pristine GF and A-GF, the NCNT-GF could offer more and efficient active sites towards Ce(IV)/Ce(III) couples, and the mechanism for the catalytic process of the cerium redox couple reaction on NCNT-GF is proposed as follows.

During the reaction process, reactant ions (including Ce^3+^ and Ce^4+^) with positive charge can be easily absorbed on the active sites, due to the relatively high negative charge density of the incorporated N with strong electronegativity [58]. The excess electrons of N can then induce the formation of localized states around N dopants [66] in NCNT-GF to form N-Ce transitional states, by matching the empty molecular orbitals of Ce^3+^ or Ce^4+^. In the subsequent catalytic process, the electrons on electrodes would transfer out of (into) the antibonding orbitals of Ce^3+^ (Ce^4+^) along the N-Ce bonds, with the positive (negative) shift of electrode potential. Thus, electron transfer between the electrode and cerium species can be facilitated by doped nitrogen which acts as an electron donor. In the final step, ion exchange occurs between the cerium ions attached to the electrode surface and the protons from the electrolyte, and the reaction products diffuse back into the electrolyte solution. Therefore, the better electrochemical properties of NCNT-GF towards cerium ion couples are due to the introduced nitrogen dopant, which create new active sites to improve the adsorption and desorption of cerium redox ions and promote ion exchange during the reaction of the Ce(IV)/Ce(III) redox couples. The fact that NCNT-GF possesses a lower O and higher N content relative to A-GF indicates that N dopants may be the catalytically active component; nevertheless, the catalytic contribution from O dopants could not be completely excluded.

**Figure 6:**
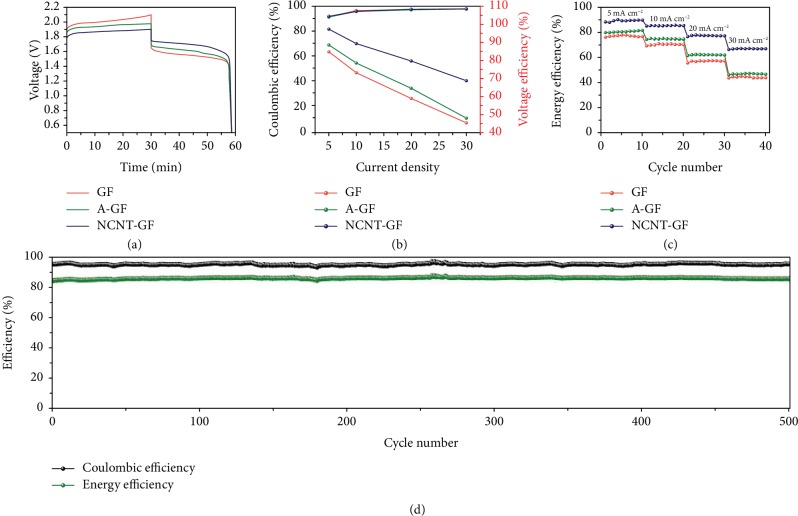
(a) Typical voltage profiles of the RFB cells assembled with GF, A-GF, and NCNT-GF. (b) CEs and VEs and (c) rate performance of cells using different electrodes. (d) Cycling performance of the RFB cell assembled with NCNT-GF at 10 mA cm^−2^.

Typical charge/discharge tests are performed with a single RFB cell to evaluate and compare the performances of graphite felt samples when used as RFB electrodes. In agreement with the aforementioned CV results, as shown in Figure 6(a), the voltage profile corresponding to NCNT-GF during the charge/discharge process demonstrates the smallest overpotentials (highest discharge voltage and lowest charge voltage) among all the samples due to the superior electrocatalytic activity of NCNTs towards cerium redox reactions. In this case, identical electrolytes, membranes, and negative electrodes are used in the cell tests. Accordingly, the overpotentials arising from ohmic and concentration polarizations may remain the same. Then, the only variation in the flow cells tested is the positive electrode surface property which could possess remarkable influence on not only the redox reaction kinetics but also the charge transfer. Consequently, any improvement in RFB performances can be because of the superior catalytic effect of NCNTs towards Ce(IV)/Ce(III) couples.

The voltage efficiencies (VEs) and coulombic efficiencies (CEs) for cells with GF, A-GF, and NCNT-GF as a function of current density are presented in Figure 6(b). It can be seen that CE values slightly increase with increasing charging/discharging rates for all samples, indicating the slow gas evolution side reaction and the minor crossover of active ions [26–28]. On the contrary, the VE is apparently lower at higher current densities because of the considerable increase in overpotential during the charge/discharge process. For the same operating current density, surface modification has little effect on the enhancement of CE values for GF electrodes. However, it directly affects the VE values, and the RFB with NCNT-GF presents much a higher VE value than those of GF and A-GF, because of the reduced cell overpotential (Figure 6(b)). This matches well with CV results where we suggest that NCNTs on the NCNT-GF surface can facilitate the sluggish Ce(IV)/Ce(III) reaction by lowering the kinetic activation energy.

Energy efficiency (EE), which is a derivative of the CE and VE, is considered as one of the most important criteria in the evaluation of RFB performance. As shown in Figure 6(c), the trend of the EE is very similar to that of the VE because of the slight fluctuation of the CE. The EEs of the RFB cells using NCNT-GF, as depicted in Figure 6(c), are dramatically superior to those of the cells using A-GF and GF, especially at high charge/discharge rates. The cell assembled with NCNT-GF affords the considerably higher EE of 89.4% relative to the RFB cells with A-GF (80.7%) and pristine GF (77.6%) when 5 mA cm^−2^ is used. Even at a high rate of 30 mA cm^−2^, the EE of the cell assembled with NCNT-GF can still reach a noticeably enhanced value of 67.6%, which is about 43.8% and 53.4% more efficient than the cells with A-GF and pristine GF, respectively. The outstanding electrochemical performance of the cell employing NCNT-GF could originate from the excellent catalytic effects introduced by the nitrogen-doped CNTs with abundant active sites, which would accelerate charge transfer and diffusion kinetics.

Some graphite-based electrodes show poor cyclability over the long-term operation owing to the erosion of the graphite surface [67]. This problem still remains a huge challenge in the development of suitable electrode materials for RFB applications. Considering this reason, we employ CNTs which have been confirmed to possess much more erosion resistance than carbon fiber and graphite [68, 69] to modify the GF, in order to protect the GF surface and provide more active sites. To evaluate the erosion resistance of the NCNT-GF electrode, Figure 6(d) plots the changes in CE and EE versus the charge/discharge cycles for the NCNT-GF. No obvious reduction in CE and EE can be observed over the consecutive 500 charge/discharge cycles, demonstrating the excellent chemical and electrochemical robustness of the NCNT-GF electrode under the harsh acidic and oxidizing conditions. Furthermore, the NCNT-GF shows no noticeable changes in their morphologies even after the 500 times of charging/discharging cycling ([Supplementary-material supplementary-material-1]). The result further confirms the erosion resistance capability of the NCNTs and the stable conjunction of NCNTs with carbon fibers of the GF.

## 3. Conclusion

In summary, NCNTs have been directly grown on the GF surface, through a scalable direct thermal pyrolysis strategy, simply using a common tube furnace. The as-prepared NCNT-GF exhibits excellent electrochemical activity towards Ce (IV)/Ce (III) redox couples, owing to the fast charge transfer and mass transfer rates allowed by the abundant N-containing functional groups. As a result, the cell with the obtained NCNT-GF serving as the positive electrode demonstrates dramatically enhanced EE values by approximately 43.8% and 53.4%, respectively, at a high current density of 30 mA cm^−2^ compared to those achieved with the A-GF and GF electrodes. Therefore, the NCNT-GF electrode possesses excellent application prospect in cerium-based RFBs. Furthermore, the synthesis strategy presented in this work could be extended to the preparation of other heteroatom- (e.g., phosphorus, boron, and sulfur) doped CNT/GF hybrids as advanced electrode materials towards RFB batteries with high performance. Also, the syngenetic strategy proposed in this work still possesses considerable potential for advancement. For example, it is well-known that the diameter and aspect ratio of individual CNTs play a critical role in determining the CNT properties [70–72]. Therefore, it is possible to optimize these characteristics to improve the catalytic activity of CNTs and thus the cell performance in the future work, through controlling the growth conditions (e.g., size of Ni catalysts and the temperature applied in the pyrolysis process) [73].

## 4. Materials and Methods

### 4.1. Preparation of Electrodes

The NCNT-modified graphite felt was prepared *via* a one-step catalyst-assisted growth approach where pyrrole was used as the nitrogen and carbon sources (see [Supplementary-material supplementary-material-1] in Supplementary Materials for detail regarding the device fabrication). Typically, a piece of graphite felt (GF), with a dimension of 3.0 cm × 6.0 cm was ultrasonically cleaned with acetone and dried at 70°C to remove the possible impurities prior to the surface modification. After that, the GF was immersed into the as-prepared nickel nitrate solution in acetone (100 mL, 3 wt%) and dried at 80°C for 5 h. To grow the NCNT catalyst on GF, the nickel nitrate deposited GF and 2.0 mL pyrrole were put into two separated ceramic boats in a quartz tube with pyrrole at the upstream side of the tubular furnace ([Supplementary-material supplementary-material-1]). Subsequently, the sample was heated to 800°C for 2 h with a heating speed of 10°C/min in a static Ar/H_2_ (5% H_2_) atmosphere and then naturally cooled to ambient temperature. The resultant sample was immersed into concentrated sulfuric acid at 80°C for 4 h to remove metal impurities. After being thoroughly washed with deionized water, the sample was dried at 80°C in air for 12 h and denoted as NCNT-GF. For comparison, pristine graphite felt sample was also treated with concentrated sulfuric acid at 80°C for 4 h as a control sample and denoted as A-GF.

### 4.2. Material Characterization

A Hitachi S-4800 field emission scanning electron microscope (SEM) was used to characterize the morphologies of samples. The Raman spectra were collected with a customized LabRAM HR800 confocal Raman microscope (HORIBA Jobin Yvon). Transmission electron microscopy (TEM) and high resolution transmission electron microscopy (HRTEM) images acquired on a FEI Tecnai G2 S-Twin were used to determine size, interlayer spacing, and sample morphology. Surface elemental analysis was performed by X-ray photoelectron spectroscopy (XPS) using an ESCALABMKLL X-ray photoelectron spectrometer.

### 4.3. Electrochemical Measurements

Electrochemical measurements were conducted by using a Bio-Logic VMP3 electrochemical workstation with reference to saturated Ag/AgCl electrodes and large GF strips as the counter electrodes. The pristine and as-prepared electrodes with geometric surface areas of 1.0 cm^2^ were chosen as the working electrodes. The electrochemical behavior of the electrode samples was investigated in 0.05 M Ce(III) methanesulfonate + 1.0 M methanesulfonic acid (MSA) electrolyte solutions. Cyclic voltammogram (CV) was run varying from 3 mV s^−1^ to 10 mV s^−1^ between the potential limits of 0.8 V and 1.8 V. Electrochemical impedance spectroscopy (EIS) for different samples were recorded in a frequency range of 10^5^ Hz to 10^−2^ Hz at a polarization potential of 1.50 V.

For the RFB cell experiments, we adapted the cell test system (NEWARE, 5 V/1 A) with an in-house designed RFB cell with an exposed electrode area of 8 cm^2^ (4.0 cm × 2.0 cm). A piece of graphite felt and a graphite plate were served as the positive and negative active electrodes, respectively. A GEFC-104 membrane was used as a proton-exchange membrane. The initial negative electrolyte used 15 mL of 1.5 M Pb(II) methanesulfonate and1.0 M MSA, and the initial positive electrolyte used 15 mL of 1.0 M Ce(III) methanesulfonate and 1.0 M MSA. This single RFB cell was charged to a previously determined capacity of 40 mA h and then discharged to 0.5 V at constant current densities.

## Supplementary Materials

Supplementary Materials Figure S1: photograph of the set-up used for the NCNT growth. Figure S2: (a) SEM image and (b–g) EDS elemental mapping images of the nickel nitrate-coated GF. Figure S3: TEM image of the NCNT before acid treatment. Residual metal nanoparticles can be observed. Figure S4: CV curves of the (a) GF, (b) A-GF, and (c) NCNT-GF electrodes in 0.05 M Ce(III) methanesulfonate + 1.0 M MSA electrolyte at various scan rates. Insets: the plot of the peak current *vs.* the square root of each scan rate. (d) Comparison of standard rate constant (*k*_0_) for GF, A-GF, and NCNT-GF. Figure S5: SEM images of the NCNT-GF electrode after 500 charge/discharge cycles. Table S1: surface species content of different samples by XPS results (%). Supplementary Methods: the methods consist of the following: Calculation of S1: The electrochemical surface area (ECSA) and S2: the standard rate constant, *k_0_*.
