# Supplementary material for: General Growth of Carbon Nanotubes for Cerium Redox Reactions in High-Efficiency Redox Flow Batteries
Source: Research (Wash D C). 2019 Nov 11;2019:3616178. doi: 10.34133/2019/3616178 (PMC6946258; doi:10.34133/2019/3616178)
Supplement: Supplementary Materials — Figure S1: photograph of the set-up used for the NCNT growth. Figure S2: (a) SEM image and (b–g) EDS elemental mapping images of the nickel nitrate-coated GF. Figure S3: TEM image of the NCNT before acid treatment. Residual metal nanoparticles can be observed. Figure S4: CV curves of the (a) GF, (b) A-GF, and (c) NCNT-GF electrodes in 0.05 M Ce(III) methanesulfonate + 1.0 M MSA electrolyte at various scan rates. Insets: the plot of the peak current vs. the square root of each scan rate. (d) Comparison of standard rate constant (k0) for GF, A-GF, and NCNT-GF. Figure S5: SEM images of the NCNT-GF electrode after 500 charge/discharge cycles. Table S1: surface species content of different samples by XPS results (%). Supplementary Methods: the methods consist of the following: Calculation of S1: The electrochemical surface area (ECSA) and S2: the standard rate constant, k0. [file 3616178.f1.docx]

**Supplementary Materials**

Title

General Growth of Carbon Nanotubes for Cerium Redox Reactions in High-Efficiency Redox Flow Batteries

Short Title

General Growth of Carbon Nanotubes for Redox Flow Batteries

**Authors**

Zhaolin Na^1^*, Ruifang Yao^1^, Qing Yan^1^, Xudong Sun^1,2^*, and Gang Huang^3^*

**Affiliations**

^1^Liaoning Engineering Laboratory of Special Optical Functional Crystals, College of Environmental and Chemical Engineering, Dalian University, Dalian 116622, China

^2^Institute of Ceramics and Powder Metallurgy, School of Materials Science and Engineering, Northeastern University, Shenyang, Liaoning 110819, China

^3^WPI Advanced Institute for Materials Research, Tohoku University, Sendai 980-8577, Japan

Correspondence should be addressed to Xudong Sun; xdsun@mail.neu.edu.cn, Gang Huang; huang.gang.e5@tohoku.ac.jp and Zhaolin Na; nazhaolin@dlu.edu.cn.


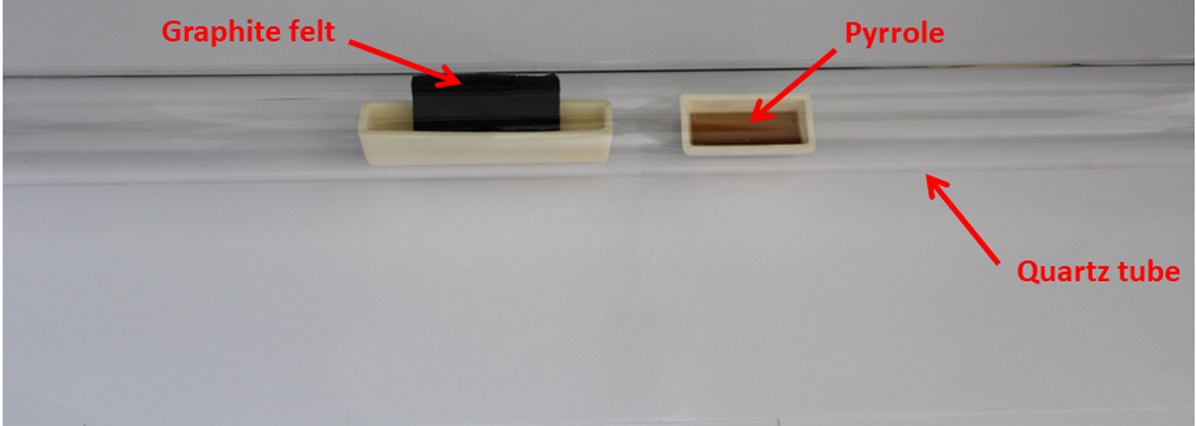


**Figure S1:** Photograph of the set-up used for the NCNT growth.


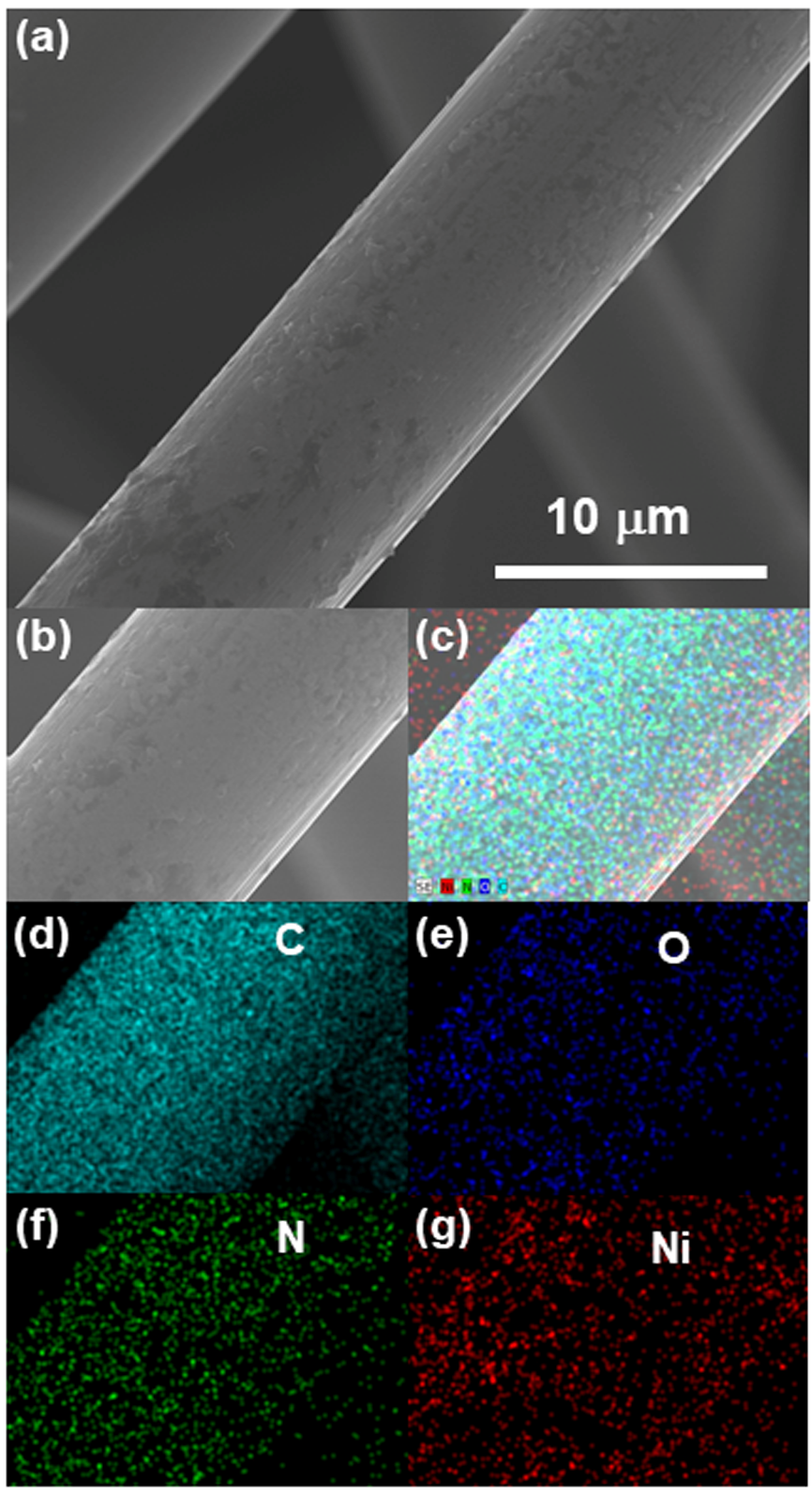


**Figure S2:** (a) SEM image and (b-g) EDS elemental mapping images of the nickel nitrate-coated GF.


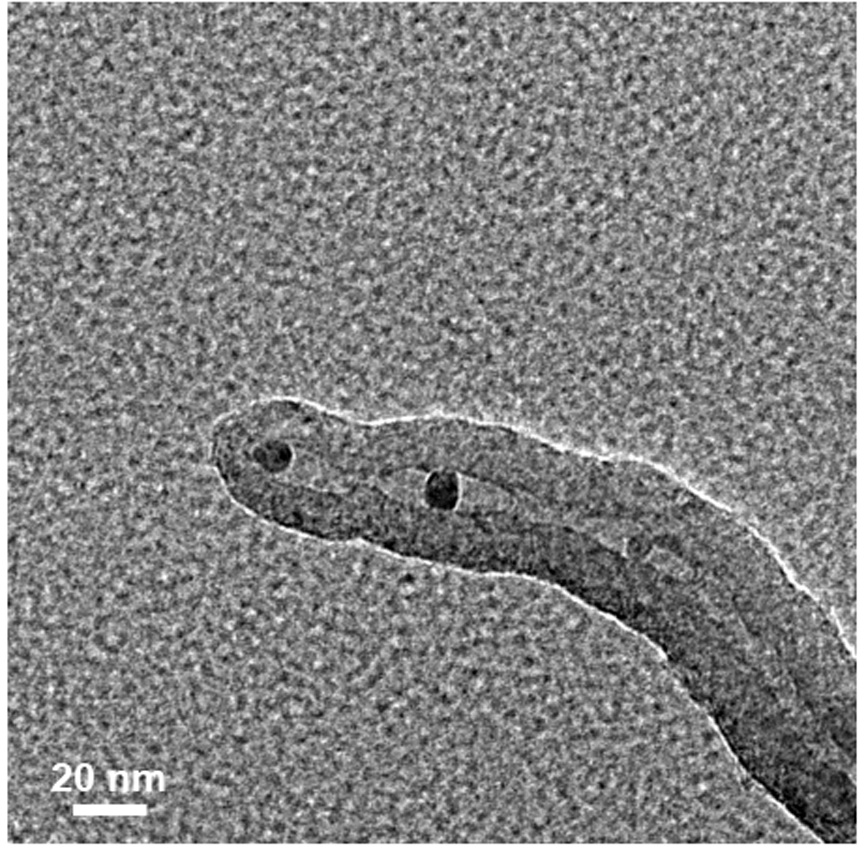


**Figure S3:** TEM image of the NCNT before acid treatment. Residual metal nanoparticles can be observed.

| **Table S1** –Surface species content of different samples by XPS results (%). | | | | |
| --- | --- | --- | --- | --- |
| Samples | C | O | N | Ni |
| GF | 92.14 | 7.86 | N.D. | N.D. |
| A-GF | 88.52 | 11.48 | N.D. | N.D. |
| NCNT-GF | 86.85 | 8.25 | 4.90 | N.D. |

*N.D. – not detectable


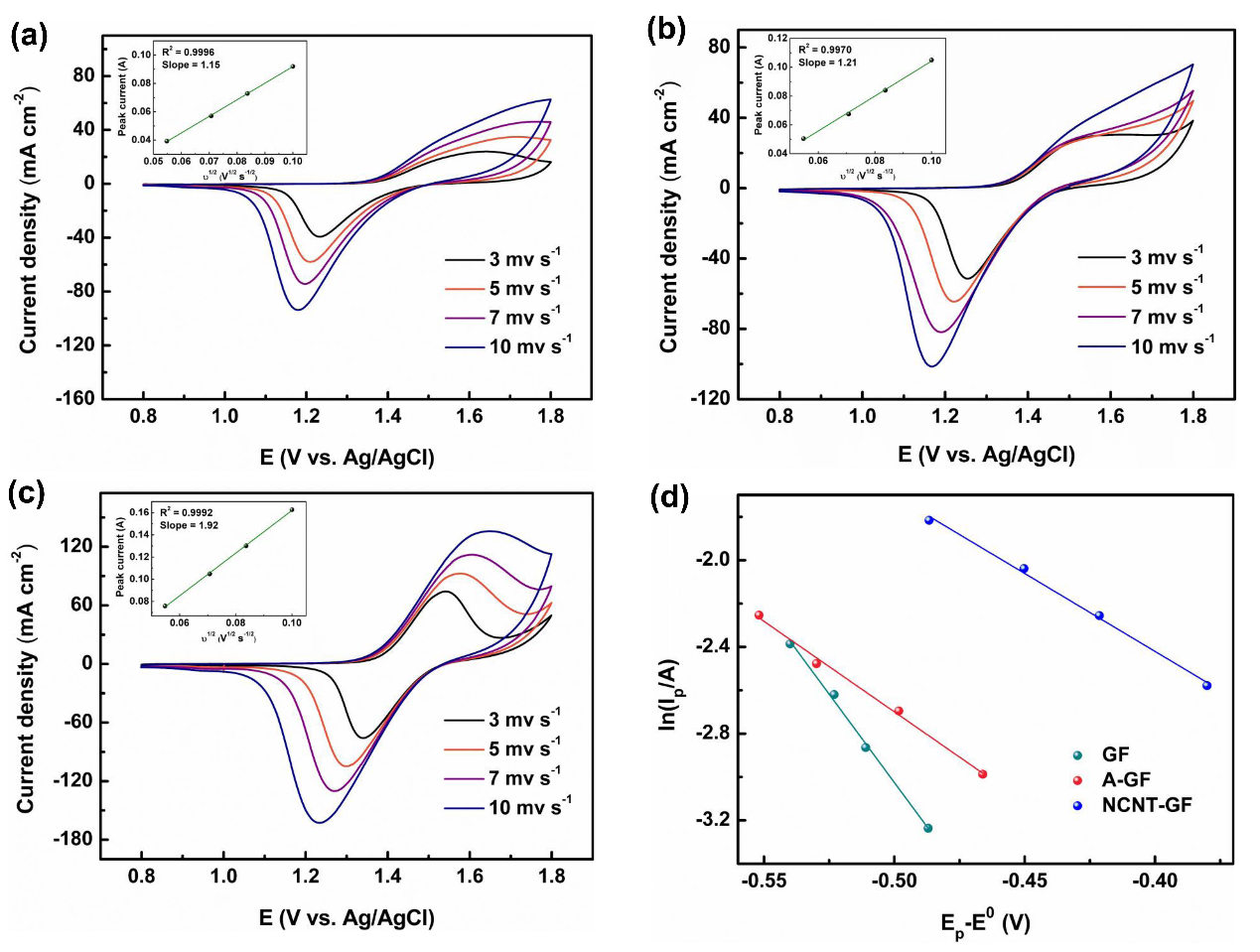


**Figure S4:** CV curves of the (a) GF, (b) A-GF and (c) NCNT-GF electrodes in 0.05 M Ce(III) methanesulfonate + 1.0 M MSA electrolyte at various scan rate. Insets: the plot of the peak current *vs.* the square root of each scan rate. (d) Comparison of standard rate constant (*k_0_*) for GF, A-GF and NCNT-GF.

**S1. The electrochemical surface area (ECSA)**

The electrochemical surface area (ECSA) can be estimated from the Randles-Sevcik equation, what relates the peak current with the square root of the scan rate, as follows:

$$i_{p}=2.99\times10^{5}n\left(\alpha n\right)^{1/2}AD_{0}^{1/2}C_{0}\nu^{1/2}$$

where *i_p_* is the peak current, *n* is the number of electrons involved in the electrode reaction, *α* is the transfer coefficient (0.5), *A* is the ECSA (cm^2^), *C**_0_* is the concentration of the electroactive species in the bulk solution (mol cm^-3^), *D_0_* is the diffusion coefficient (cm^2^ s^-1^) and *v* is the scan rate (V s^-1^). The values of the diffusion coefficients were obtained from bibliography, 0.69×10^−6^ cm^2^ s^−1^.^1^

**S2. The standard rate constant, k_0_**

The standard rate constants can be determined using the following equation:

$$i_{p}=0.227nFAC_{0}k_{0}\exp\left[\frac{-\alpha nF\left(E_{p}-E^{0}\right)}{RT}\right]$$

where *i_p_* is the peak current, *n* is the number of electrons involved in the reaction, *F* is the Faraday constant, *A* is the active surface area of the electrode, *C_0_* is the bulk concentration of the electroactive species, *α* is the transfer coefficient (0.5), *E_p_* is the peak potential, *E^0^* is the formal potential, *R* is the gas constant and *T* is the electrolyte temperature. Therefore, a plot of ln (*i_p_*) *vs.* *E_p_-E^0^* for different scan rates, should yield a straight line with a slope of *-αnF/(RT)* and an intercept proportional to *k_0_*. Fig. S2d depicts the plots of ln (*i_p_*) *vs.* *E_p_-E^0^* obtained from the CV data, from which *k_0_* values can be calculated.


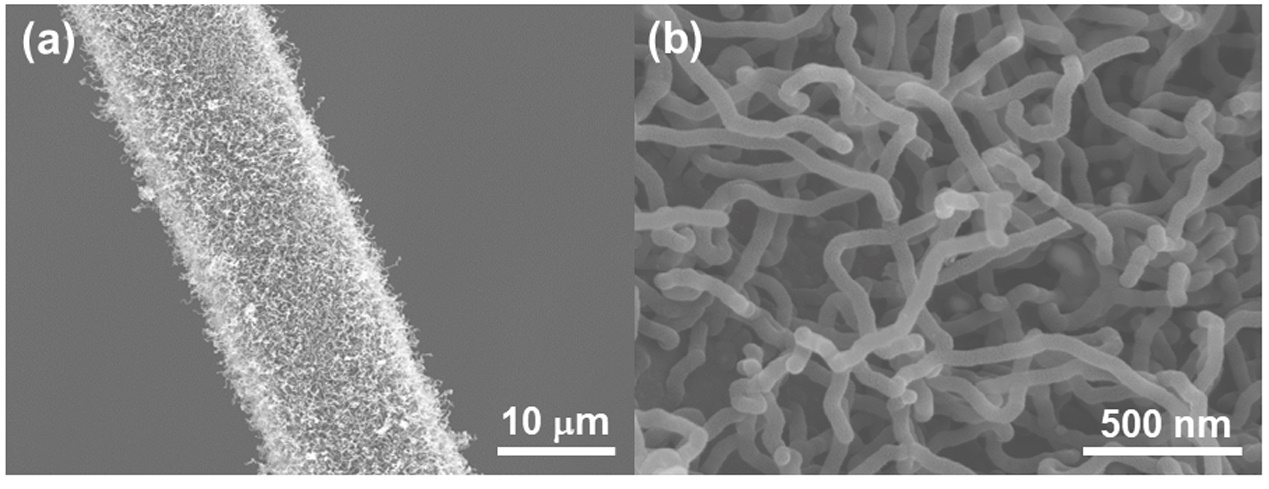


**Figure S5:** SEM images of the NCNT-GF electrode after 500 charge/discharge cycles.

**Supplementary Reference**

(S1) P. K. Leung, C. Ponce-de-León, C. T. J. Low and F. C. Walsh, *Electrochim. Acta*, 2011, **56**, 2145-2153.
